# The various insecurities experienced among non-standard workers across different policy-contexts: Emilia Vignola

**DOI:** 10.1093/eurpub/ckac130.126

**Published:** 2022-10-25

**Authors:** K Bosmans, E Vignola, V Álvarez López, M Julià Pérez, S Baron, T Bodin, PWR consortium

**Affiliations:** 1 Interface Demography, Vrije Universiteit Brussel, Brussels, Belgium; 2 Community Health and Social Sciences, City University of New York School of Public Health, New York City, USA; 3 School of Sociology, University of Valparaíso, Valparaíso, Chile; 4 SDHEd, Hospital del Mar Medical Research Institute, Barcelona, Spain; 5 ESIMar, Parc de Salut Mar, Barcelona, Spain; 6 GREDS-EMCONET, Universitat Pompeu Fabra, Barcelona, Spain; 7 Commoner Center Health and the Environment, Queens College, City University of New York, New York City, USA; 8 Unit Occupational Medicine, Karolinska Institutet, Stockholm, Sweden; 9 Centre for Occupational and Environmental Medicine, Stockholm Region, Stockholm, Sweden

## Abstract

**Background:**

Over the last decades, the prevalence of non-standard employment (NSE) has increased in many countries, with negative implications for worker health and well-being. Research at the micro level, mostly quantitative, has linked NSE with poor health through insecurity. Macro-level studies investigating whether political economic factors buffer the harms of NSE have generated mixed results. This study describes how various types of insecurity are experienced by workers in NSE, in general and during COVID-19, and how this influences their health and well-being, in six countries with different welfare states: Belgium, Canada, Chile, Spain, Sweden and the United States.

**Methods:**

In-depth interviews with 250 workers in NSE were analysed using a multiple-case study approach and using the welfare state typology as a macro-level framework.

**Results:**

Despite differences in welfare states, workers in all six countries experienced multiple forms of insecurity as well as relational tension with employers or clients, with clear negative effects on their well-being, in ways that were shaped by broader social inequalities (e.g., related to gender, age, and access to family support). Simultaneously, differences in welfare states were reflected in the level of workers’ exclusion from social protections, the temporality of difficulties they faced in planning their lives (e.g., threats to daily survival or to longer-term life planning), and their ability to derive control from NSE despite the insecurity it created. Workers in less generous welfare states experienced heightened insecurity and greater stress from the COVID-19, but the severity of the health and economic crisis was felt by workers in all study countries.

**Conclusions:**

This study sheds light on the ways that welfare regimes can support - or fail to support - workers in NSE, and suggests the need in all six countries for stronger state responses to NSE, a pressing social determinant of health.

**Key messages:**

- Employment insecurity is a central dimension of the experience of non-standard employment across the six countries.
- Differences in state approaches to labour market regulation and social welfare influence how non-standard workers experience insecurities.

